# Acute systemic inflammatory response after cardiac surgery in patients infected with human immunodeficiency virus using clinical and inflammatory markers

**DOI:** 10.4314/ahs.v17i3.14

**Published:** 2017-09

**Authors:** Mawande KE Gojo, Rosaley Prakaschandra

**Affiliations:** 1 Port Elizabeth Hospital Complex, Department of Cardiothoracic Surgery, Buckingham Road, Port Elizabeth, South Africa. Gojomawande@yahoo.com; 2 Durban University of Technology, Department of Biomedical and Clinical Technology; 41/43 ML Sultan Road, Durban, South Africa. Rosaleypra@dut.ac.za

**Keywords:** Cardiopulmonary bypass (CPB), Human immunodeficiency virus (HIV), inflammation

## Abstract

**Background:**

Immediate post-cardiopulmonary bypass (CPB) immune responses and organ injuries in immune-compromised patients remain poorly documented.

**Method:**

Sixty-one consecutive patients (30 HIV seropositive and 31 seronegative), undergoing elective cardiac valve(s) replacement were enrolled, from a single center hospital, after informed consent was obtained. C-reactive protein (CRP) and Erythrocyte sedimentation rate (ESR) were used as biomarkers of acute inflammatory response.

**Results:**

The mean age was similar between the HIV seropositive and negative group. Pre-operatively, CRP (p=0.388) and ESR (p=0.817) were comparable. The CPB events and durations were significantly different between the two groups: duration (p=0.021), clamp aortic duration (p=0.026), bloodtransfusion (p=0.013), total urine output (p=0.035) and peak lactate (p=0.040). Post-operatively, there was significant increased biomarkers level in both groups, albeit not between the groups with a significant negative correlation between the mean change in CRP levels and mechanical ventilation (r=0.548, p=0.002) in the seropositive group (r=0.025, p=0.893). The correlation between pre-operative and post-operative difference in CRP and ICU stay was not significant in both groups. A significant drop (p=<0.001) in CD4 cells was documented post-operatively in the HIV seropositive group.

**Conclusion:**

HIV positive patients' post-operative reactions to cardiac surgery supported by CPB are similar to those of HIV seronegative patients.

## Introduction

In the era of the Human immunodeficiency virus (HIV) pandemic, there have been various concerns about the operative risks, infections and benefits of cardiac surgery in patients infected with HIV, as they are known to already have a compromised immune system, chronic inflammation and reduced survival.^1^,^2^ This led to cardiac surgery departments setting a minimum acceptable CDT T-cell count level for HIV positive patients to undergo elwctive corrective cardiac surgery, ranging between 250 and 400 cells/cm^3^. Acquired immunodeficiency syndrome (AIDS) is diagnosed when the CD4 T-cells level count drops below 200 cells/cm3 or when opportunistic infections arise.^3^

Following the introduction of highly active antiretroviral treatment (HAART), the lifespan of HIV positive patients has increased dramatically. In addition, recent research studies suggest that little difference exists with regards to surgical outcomes and duration of hospital stay between HIV positive patients and negative patients undergoing cardiac surgery.^4^

There is very limited information published prospectively on the HIV disease profile in correlation with alterations and immune system activation during and after cardiac surgery supported by CPB. The aim of the study was to investigate the severity of acute systemic inflammatory response after cardiac surgery in patients infected with human immunodeficiency virus in comparison with HIV negative patients, using C-reactive protein (CRP) and Erythrocyte sedimentation rate (ESR) biomarkers. This study also sought to identify pre-operative risk and peri-operative complications to determine whether clinical relevant events correlate to alterations of immune responses and organ injuries based on laboratory tests

## Materials and methods

### Participants

A total of sixty-one (61) participants were selected as participants from a single centre hospital, Inkosi Albert Luthuli Central Hospital (IALCH) and were divided into two groups i.e. Thirty HIV positive patients and thirty-one HIV negative patients undergoing elective on-pump cardiac surgery. These HIV positive patients were recruited consecutively, and a relatively corresponding age-and-gender matched ‘control’ HIV negative patient was selected, after informed consent had been sought. After noting that no prospective similar study has been published, because most studies on HIV patients were retrospective studies, case reports and literature reviews, a biostatistician was consulted to verify the number of participants and data variables required to significantly test the hypothesis.

### Materials

A Stockert S5 heart - lung machine (CPB) utilizing roller pump was used in all patients during data collection. Eurosets Admiral oxygenator and tubing was the only oxygenator used during data collection. Admiral oxygenator internal surfaces are coated with Phosphorylcoline which is the predominant lipid head-group found in the outer layer of cell membranes. Phosphorylcoline has a natural affinity for water and binds water tightly around itself, as a result does not promote clots formation. Admiral oxygenators require a priming volume of 190 ml, have a contact surface area of 1.35 meter square with maximum blood flow rate of 7.0 litre/min. Total CPB circuits priming solution included 1450ml of Plasmalyte B, 5000i.u of heparin and 50ml of 20% Albusol. Standard protocol for CPB conduction was not modified; electrolytes and blood ph corrections were corrected as per the standard protocol to maintain normal ranges. Also, the use of adrenaline and other routine drugs were not prohibited nor limited during data collection.

Mistral cardioplegia delivery sets were used to administer cardioprotective solutions which was either cold blood cardioplegia delivered at the ratio of 4:1 blood to medsol cardioplegic solution at an average delivery pump rate of 240ml/min for the duration of four minutes induction and 2 minutes maintenance, or ST Thomas II crystalloid cardioplegic solution which was delivered at a standard protocol of 20ml/kg induction and 10ml/kg maintenance. Patients' temperatures were cooled down as per the standard unit protocol of hypothermia which for heart valvular surgery range between 28–32 °C, and then rewarmed to a minimum core temperatures of 35 °C before termination of CPB. Topical hypothermia which employs pouring ice cold normal saline on the heart was employed in all patients as a standard protocol during cardiac surgery.

ADVIA chemistry systems Wide Range C-reactive protein (wrcrp) method was used to test for CRP. This method measures CRP in serum and plasma by a latex enhanced immune-tirbidimetric assay. It is based on the principle that the analyte concentration is a function of the intensity of scattered light caused by the latex aggregates. The latex particles coated with anti-CRP rapidly agglutinate in the presence of C-reactive protein-forming aggregates. The wrcrp latex reagent is a suspension of uniform polystyrene latex particles coated with anti-CRP antibody. When serum containing CRP is mixed with the latex reagent, agglutination takes place resulting in an increase in the turbidity. This turbidity is measured at 571 nm. The CRP concentration in serum is determined from a calibration curve that is generated with calibrators.

HIV positive patients were tested for CD4 T-cell count using Panleucogating (PLG)/CD4 method which uses a sequential gating strategy to identify all CD45^+^ leucocytes in order to measure the CD4% of lymphocyte and absolute CD4 count. Absolute CD4 counts are derived by single platform analyses that incorporate the use of Flow-Count beads with a pre-defined concentration.

### Procedure

On the eve of the operation each participant was identified, recruited and asked to sign the consent if they were willing to participate in the research study. On the day of the operation upon arrival in operation room, routine invasive and non-invasive heamodynamic monitoring devices such as peripheral drip line, electrocardiogram electrode, pulse oximeter, invasive blood pressure, central venous pressure, urine catheter and temperature monitoring cable were inserted and/or placed on the patient.

### Pre-operative data

Patient demographics such as gender, age, type of surgery, cardiac ejection fraction, New York Heart Association (NYHA) classification and current medication were recorded on the data sheet.

After all monitoring devices were placed on the patients just before initiation of anaesthesia, arterial blood samples were taken from the radial or femoral invasive arterial catheter. Blood samples were collected for laboratory tests using appropriate recommended sample collection method and test tubes for each of the tests i.e. C-reactive protein, Erythrocyte sedimentation rate, and CD4 cell count (HIV positive patients only). These variable tests served as baselines

### Intra-operative data

Intra-operative samples included: myocardial protection technique employed for each case, blood products transfused, peak-lactate level, total urine output, aortic cross-clamp duration and CPB duration.

### Post-operative data

Post-operative samples included clinical assessments such as: duration on mechanical ventilation, duration on positive inotropic support and duration in the intensive care unit, all measured in days.

On the second post-operative day (48hours) blood samples were taken by the researcher for post-operative laboratory tests i.e. C-reactive protein, Erythrocyte sedimentation rate and CD4 cell count (HIV positive patients only). These samples were taken according to each test standard recommended time that it reach peak levels at, and in this study all variables under study are categorized as reaching peak after 48 hours of surgery.

Patients were followed-up until they were extubated, weaned off inotropes and transferred out of intensive care unit.

Microsoft excel was used to record variables and related demographics for statistical analysis. These were then transferred to SPSS version 23 for further analysis.

**Ethical considerations:** This study was granted ethical approval from the Durban University of technology Institutional Research and Ethics committee (Chairperson: Prof JK Adam, IREC ref no: REC 96/14, 05/05/2015).

## Results

There was a dominance of females (54.1%), with 45.9% being males. The majority of participants (36.1%) were seen in the 30–40 year age group (Table 1).

**Table 1:** Clinical characteristics of the study and control population.

Variables	Study group (HIV positive)/n=30	Control (HIV negative) /n=31
Female	16 (53%)	17 (55%)
Male	14 (47%)	14 (45%)
Age (mean ±)	37.8 ± 10.6	37.1 ± 11.9
Aortic valve Replacement	8 (27%)	4 (13%)
Mitral valve Replacement	19 (63%)	10 (32%)
Double valve Replacement	3 (10%)	17 (55%)
NYHA grading: I II III IV	2 (7%) 21 (70%) 6 (20%) 1 (3%)	2 (6%) 18 (58%) 8 (26%) 3 (10%)
Ejection fraction (mean):	48.6% ± 9.4	51.1% ± 11.2
Type of cardioplegia: ST Thomas II Blood 4:1	18 (60%) 12 (40%)	23 (74%) 8 (26%)
HAART: Yes No	26 (86.7%) 4 (13.3%)	---- ----
Mortality:	1 (3.3%)	1 (3.2%)

* Clinical Characteristics of the study and control population

The mean age for the HIV seropositive was 37.8 ± 10.6 and 37.1 ± 11.9 years for the HIV seronegative group with the range being between 18 to 60 years. Types of operations comprised of aortic valve replacement (AVR) 19.7%, mitral valve replacement (MVR) 32.8% and double valve replacement (DVR) 47.5%, which is a combined operation for both AVR and MVR. Baseline New York heart association (NYHA) of all patients grading was statistically analyzed to determine the frequency for each grade. Grade I frequency was four patients (6.6%), grade II frequency was thirty-nine patients (63.9%), grade III frequency was fourteen (23.0%) and grade IV frequency was four (6.6%). The baseline pre-operative ventricular ejection fraction (EF) was analyzed and compared between the two groups, with the mean EF of 48.6% in the study group, and 51.1% in the control group. Two types of cardioprotective cardioplegia solution were used in the study; with the type of cardioplegia was to be employed being determined by the surgeon. Twenty (32.8%) patients used Buckberg blood 4:1 cardioplegia and forty-one (67.2%) used ST Thomas II crystalloid cardioplegia. Out of thirty patients in the study group, twenty-six (86.7%) were on HAART, and four (13.3%) were not on any anti-retroviral treatment. The total study mortality from the sixty-one patients was two patients (3.3% mortality). Only one patient did not complete the study follow up period, as the patient died three hours after the operation (3.3% mortality in the HIV positive group). This patient could not be excluded from the data since the cause of death may be related to the stratified operative risks under study. All other sixty patients completed the follow up period, with one other HIV negative patient dying later while still in hospital (3.2% mortality in the HIV negative group).

**Figure 1:**
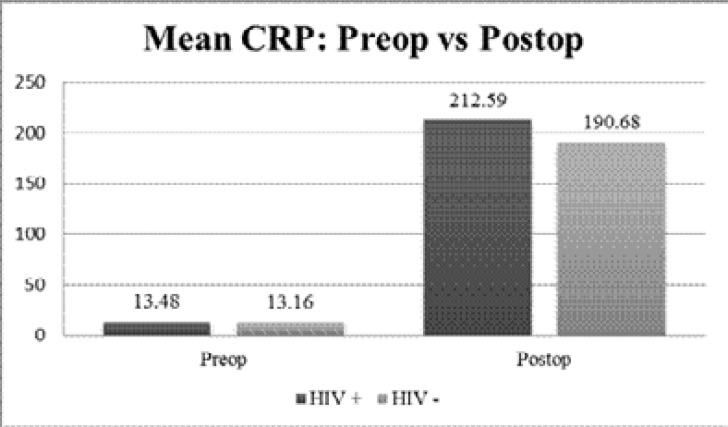
Graphical comparison of mean CRP (C-reactive protein) levels pre and post-operatively between the two groups

The pre-operative CRP analysis between the study and control group showed no significant difference (p=0.388 and 0.817, respectively) (Figure 1).

**Figure 2:**
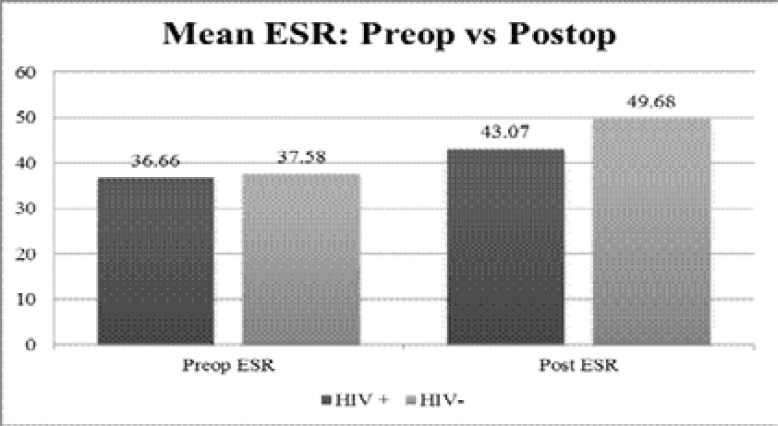
Graphical representation of mean ESR levels pre and post-operatively between the two groups

Post-operatively CRP mean was slightly higher in the study group (212.59 ± 23.731), as compared to the control group (190.68 ± 57.919), however statistical analysis showed that the difference in results was not significant (p=0.115). Similarly, the mean ESR between the study group (43.07 ± 23.067) and control group (49.68 ± 19.139) showed no significant difference (p=0.214) (Figure 2).

To determine if there were predisposing factors (viral load, CD4 count and anti-retroviral medication) that could increase risks of cardiac surgery supported by CPB in HIV positive patients, patients infected with HIV were investigated to evaluate the changes in these parameters (Table 2). The pre-operative CD4 cells levels were compared with levels at three days post-operative. Paired sample statistical results showed pre-operative CD4 cells mean of 598.76 ± 205.576 and the post-operative CD4 cells mean was 405.97 ± 119.089.

**Table 2:** Correlation of predisposing factors and ICU stay

Parameters	*r* - value	*p*-value
CD4 cell diff vs. CPB duration	.258	.177
CD4 cell diff vs. Age	.064	.741
CD4 cell diff vs. NYHA grading	.056	.775
CD4 cell diff vs. ICU stay	.248	.195

As anticipated, there was a decrease in CD4 cells post-operatively, and this difference was strongly significant (p= <0.001) when compared to pre-operative levels. The difference between preoperative and postoperative CD4 cells levels was further analyzed and correlated with surgical outcome determinant variables such as CPB duration, age, NYHA grading and ICU stay to investigate whether or not CD4 cells level is a predictive factor for surgical outcome in HIV positive patients.

**Figure 3:**
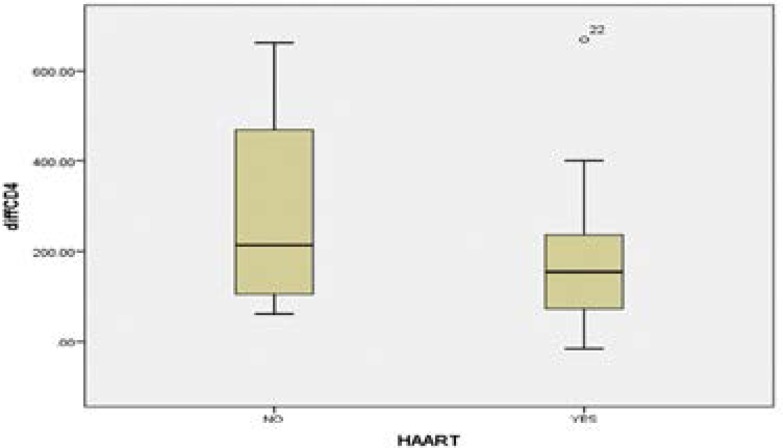
Difference in CD4 cells between patients on HAART and those who are not

There was no significant correlation between change in CD4 cells and CPB duration, age, NYHA grading and ICU stay (p=0.177, 0.741, 0.775 and 0.195 respectively). In this study only the highly active anti-retroviral treatment proved to be a predictive factor for CD4 cells level changes after cardiac surgery in these immune-compromised patients (Figure 3). The group of patients on HAART had the CD4 cells difference mean of 177.7600 ± 151.36, as compared to the mean of 286.7500 ± 264.76 in patients who were not taking anti-retroviral treatment.

## Discussion

Cardiac surgery and cardiopulmonary bypass (CPB) initiates a systemic inflammatory response, resulting in a whole-body inflammatory reaction.^5^ The extent of this inflammatory reaction will vary from patient to patient, with persistence of any degree of inflammation considered potentially harmful to the cardiac patient.^6^, and may further be exacerbated in patients infected with HIV leading to increased morbidity and mortality.^7^ In the era of the HIV pandemic, studies have earlier reported poor outcomes of cardiac surgery in HIV seropositive population.^8^,^9^ As a result cardiac surgery in HIV seropositive patients was performed with caution and forbidden in patients with AIDS, due to fear of post-operative risks of infection.^2^,^10^

With the introduction of highly active antiretroviral treatment (HAART), studies thereafter have reported excellent surgical outcomes in HIV seropositive patients.^11^ As the number of HIV positive patients requiring open heart surgery increases, and is expected to increase further as the incidence increases, it becomes imperative to understand the effect of cardiac surgery with CPB in HIV positive subjects. Furthermore, there is a paucity of data related to the South African context, which our study has been tempted to address. To the knowledge of the authors this makes this study the first and largest prospective study to be ever conducted in HIV positive patients undergoing cardiac surgery supported by CPB in South Africa.

Our study reports no post-operative complications, as well as a very low overall mortality, as out of sixty-one patients, only two patients (3.3%) demised (one patient in each group). In both demised patients, pre-operative and post-operative variables were within the same ranges as other patients, and there was no variable or biomarkers that were profoundly high when compared to the remaining patients to associate with a possible predictive cause of death.

Our study also reports an absence of post-operative, acute renal failure requiring dialysis, neurological complications and sepsis in both groups, including HIV seropositive patients who were not on HAART treatment even though they are at high risk to infections.^12^ We also found that differences in pre-operative and post-operative CRP levels correlated strongly with duration of mechanical ventilation in the study group. The C-reactive protein (CRP), being a reliable indicator for the acute systemic inflammatory response, has become a necessity in the monitoring and management of cardiac patient during the preoperative and post-operative periods.^13^–^14^ Elevated preoperative CRP levels as minimal as 5mg/L have been found to be independent risk factors associated with increased risk of complications and post-operative infections.^15^ A pre-operative mean CRP of 13.48mg/L in the HIV positive group, and 13.16mg/L in the HIV negative group were found in our study, but were not associated with complications or adverse outcomes post-operatively. In addition, we did not show the pattern of HIV positive individuals having significantly high levels of CRP as reported by Bryan et al^16^.

The post-operative inflammatory responses have been found to influence the development of multiple organ failure including myocardial dysfunction, respiratory failure and renal dysfunction.^17^ In our study, as expected, CRP levels increased significantly post-operatively in both HIV positive and negative groups, but were similar. However, significant differences were observed between the two groups when CRP levels were correlated with postoperative clinical outcomes. A strong negative correlation between the CRP levels and post-operative mechanical ventilation duration in the HIV positive group was observed, in keeping with the results from Taher et al,^18^ but was not observed in the HIV negative group.

Khaled et al^19^ reported that elevated CRP levels post-operatively are associated with a prolonged stay in the intensive care unit (ICU) and that a post-operative CRP level of about 51mg/L correlated significantly with the ICU length stay (p=0.04) duration up to 7 days.^19^ In our study, this was not observed even though post-operative CRP was much higher than 51mg/L in both groups. In addition, none of our patients required re-admission to ICU or re-intubation for mechanical ventilation after ICU discharge. Our findings may be substantiated by those from Sophie et al and Mara et al, who found that post-operative CRP levels at the time of patients discharge from ICU did not necessarily indicate any clinical post-ICU adverse outcomes.^20^–^21^

Further analysis between CRP levels, NYHA class and age was non-significant, and in keeping with the literature.^22^,^23^ This was also observed with the ESR, which is an additional inflammatory biomarker with CRP in rheumatic fever and rheumatic heart disease^24^. These findings may be attributed to the influence of anaemia and polycythaemia, rendering it more frequently abnormal, and therefore less reliable.^25^

To further determine other possible predisposing factors for observed increased post-operative CRP levels, the difference between pre-operative and post-operative CPR levels in both groups were correlated to pre-operative and CPB variables. The findings in the HIV positive group are in contrast to current literature which supports a positive correlation between CRP and NYHA class.^25^

The duration of acute systemic inflammatory response (SIR) and mechanisms that lead to the lengthening of acute SIR in patients that have been operated on under cardiopulmonary bypass have been explored.^26^ During bypass, blood transfusions induce a second insult to the systemic inflammatory response that already exists after cardiac surgery, leading to an exacerbation of the SIR.^27^ In addition, this adverse effect of transfusion, which includes immune response reactions leading to activation of the inflammatory milieu, may be more pronounced in HIV-infected patients, and may explain the correlation between CRP levels and CPB time^28^, also observed in our HIV positive patients.

Other variables like the urine output of 0.5ml/kg/hr are predictive of renal failure post CPB, and it has been found to be a lethal complication associated with multi-organ failure.^29^ Although our study indeed observed a negative correlation between post-operative CRP and total CBP urine output in both groups, these were not statistically significant.

Hyperlactatemia is common after cardiac surgery and studies have found that a lactate threshold of about 3 mmol/L at ICU admission is able to identify a population at risk of morbidity and mortality after cardiac surgery.^30^ Although these levels were also seen in our study, it was not associated with accompanying post-surgical complications. Imhof et al^31^ reported hyperlactemia in HIV positive patients, and found that it is strongly associated with anti-retroviral medication; however in our, study pre-operative baseline lactate was not measured.^31^ In our study we showed a strong significant correlation between postoperative CRP and the lactate level during CPB in the study group (HIV+), and notes the lack of published literature in this area of research.

It has been shown that CD4 cells levels determine the progression of HIV in infected individuals.^32^ In our study the post-operative CD4 cells count was significantly lower, and mirrors those reported by Alejandro, et al.^2^ Further analysis on HIV positive patients taking HAART versus those who were not on therapy, showed that the CPB duration was significantly longer in those subjects who were not on HAART therapy. The lack of strong correlations between mean CD4 cells and variables like age, baseline NYHA grading and CPB duration suggest that even though there is a significant change in CD4 cells level post-operatively, these changes are not influenced by patient pre-operative variables and CPB duration. The weak positive correlation observed between mean CD4 cells difference are similar to those of Cacala, et al who reported that CD4 counts had no relation with in-hospital outcome in HIV-positive surgical patients.^33^ Unfortunately, we were not able to establish whether or not HAART is associated with improved outcomes following cardiac surgery with CPB in HIV positive patients, due to a short post-operative follow up period.

## Conclusion

This investigation shows that the acute systemic inflammatory response in HIV positive patients is similar to that of HIV negative patients, with similar patterns of CRP levels and ESR level. To our knowledge this is the first study to be conducted prospectively in South Africa and we indeed bridged the gap between the clinical outcomes observed retrospectively by Blyth et al.^34^ We confirmed that laboratory biomarkers are significantly increased after cardiac surgery with CPB; however they do not seem to indicate any short-term complications or poor surgical outcomes. Furthermore this study has shown that cardiac surgery may be safely performed even in subjects who are not on HAART therapy with no post-operative complications compared to those on HAART. Although we found a significant drop in CD4 cells post-operatively in HIV positive patients this did not seem to affect the short-term surgical outcomes.
